# Impact of a Single 36 Hours Prolonged Fasting Period in Adults With Type 1 Diabetes – A Cross-Over Controlled Trial

**DOI:** 10.3389/fendo.2021.656346

**Published:** 2021-07-06

**Authors:** Othmar Moser, Max L. Eckstein, Alexander Mueller, Norbert J. Tripolt, Hakan Yildirim, Farah Abbas, Peter N. Pferschy, Nandu Goswami, Felix Aberer, Anna Obermayer, Thomas R. Pieber, Harald Kojzar, Caren Sourij, Martina Brunner, Tobias Niedrist, Markus Herrmann, Harald Sourij

**Affiliations:** ^1^ Interdisciplinary Metabolic Medicine Trials Unit, Division of Endocrinology and Diabetology, Department of Internal Medicine, Medical University of Graz, Graz, Austria; ^2^ Division of Exercise Physiology and Metabolism, Department of Sport Science, University of Bayreuth, Bayreuth, Germany; ^3^ Exercise Physiology, Training & Training Therapy Research Group, Institute of Sports Science, University of Graz, Graz, Austria; ^4^ Austria Clinical Institute of Medical and Chemical Laboratory Diagnostics, Medical University of Graz, Graz, Austria; ^5^ Division of Physiology, Otto Loewi Research Center, Medical University of Graz, Graz, Austria; ^6^ Division of Cardiology, Medical University of Graz, Graz, Austria; ^7^ Clinical Institute of Medical and Chemical Laboratory Diagnostics, Medical University of Graz, Graz, Austria

**Keywords:** fasting, type 1 diabetes, CGM, safety, OGTT (oral glucose tolerance test)

## Abstract

Prolonged fasting has shown beneficial effects in healthy individuals and in people with chronic diseases. In type 1 diabetes, the effect or even the feasibility of fasting is unclear. We aimed to assess the impact and safety of prolonged fasting in adults with type 1 diabetes. Glycemia was assessed during overnight fasting (12 hours) vs. prolonged fasting (36 hours) *via* an intermittently-scanned continuous glucose monitoring system. Anthropometric data, metabolic and hormonal markers were compared between both trial arms. After each fasting period, a 75 g oral glucose tolerance test was performed and plasma glucose levels and hormones were assessed. Data were compared *via* paired t-tests and mixed-model regressions (p ≤ 0.05). Twenty individuals with type 1 diabetes (7 females) with a mean ± SD age of 35 ± 11 years, body mass index (BMI) 24.8 ± 2.8 kg/m^2^ and HbA_1c_ 54 ± 7 mmol/mol were included. Hypoglycemia/hour (70 mg/dL; <3.9 mmol/L) was similar in both trial arms (12 hrs: 0.07 ± 0.06 *vs.* 36 hrs: 0.05 ± 0.03, p=0.21). Glycemic excursions during the oral glucose tolerance test were not different after the two fasting periods. Beta-hydroxybutyrate levels were higher after prolonged fasting (p=0.0006). Our study showed that people with type 1 diabetes can safely perform a 36 hours fasting period with a low risk of hypoglycemia and ketoacidosis.

**Clinical Trial Registration:**

DRKS.de, identifier DRKS00016148.

## Introduction

Reducing food availability *via* caloric restriction (fasting) was shown to improve health, improve markers of aging and ameliorate clinical outcomes in several diseases (1–3). In general, caloric restriction can be categorized into intermittent fasting and time restricted feeding (4). Intermittent fasting is defined by repeated periods with low or no energy intake that might be further classified by alternate day fasting, alternate day modified fasting and fasting or modified fasting on two days per week. Periodic fasting is characterized by fasting for two or more days. Time restricted feeding can be defined by eating patterns, which are restricted to a short (<8–10 hours) interval each day (4). Different metabolic, hormonal and cellular effects have been described to be associated with fasting (5): energy sources switch to the utilization of fatty acids and ketone bodies (6), improvements in insulin sensitivity (7, 8) and cells activate pathways improving intrinsic defenses against oxidative and metabolic stressors (9). Furthermore, it was shown that intermittent fasting is an effective life-style intervention to lower bodyweight and improve waist circumference in overweight people with type 2 diabetes (7). Since overweight and obesity in children (10) and adults with type 1 diabetes increases and equal those of the general non-diabetes population (11), the methodology of intermittent fasting might also be applied in type 1 diabetes. In people with type 1 diabetes, fasting might lower the need for exogenous insulin (12), stabilize glucose levels in a near normoglycaemic range, lower bodyweight and body mass index (BMI) and lower the total carbohydrates intake (13).

Taking these positive effects of fasting and its potential to improve cardio-vascular outcomes into account, people with type 1 diabetes do follow dietary regimens more often. Those include also strategies incorporating longer fasting periods, however, data on the impact of fasting interventions on safety, namely hypoglycaemia and diabetic ketoacidosis and the change in the insulin dose needed following a prolonged fasting period in people with type 1 diabetes are scarce. Especially, physiological ketosis needs to be separated from diabetic ketoacidosis (13): a regular blood ketone concentration is <0.3 mmol/L, ranging during nutritional ketosis from 0.5 to 3 mmol/L. During prolonged fasting (several weeks), the beta-hydroxybutyrate can increase up to 5 - 7 mmol/L (14). Diabetic ketoacidosis is associated with an absolute lack of insulin and includes blood glucose levels usually > 250 mg/dL (13.9 mmol/L) accompanied with pH levels dropping below 7.3 and/or bicarbonate levels <18 mmol/L (15).It was shown that the rate of hypoglycemia during Ramadan fasting was 23.8─29.3% depending on the type of insulin therapy (16); however, most concerning, the rate of severe hypoglycemia was 7.1% for those using multiple daily insulin injections (MDI). While hypoglycemia is a major obstacle of fasting in people with type 1 diabetes, rates of ketosis were as low as 2.5% for people on MDI during fasting, assessed in a meta-analysis including 1,699 people with type 1 diabetes (16). Moreover, assuming a change in insulin sensitivity and a potential degradation of hepatic glycogen stores during the fasting period, the question arises if bolus insulin can be applied with a regular carbohydrate-to-bolus insulin ratio at the first carbohydrate intake following the fasting period.

Therefore, the aim of this study was to assess the safety and glycemic parameters during and after prolonged fasting in adults with type 1 diabetes on the short term as a basis for potential future longer studies of intermittent fasting regimens.

## Methods

This was a single center, cross-over controlled clinical trial assessing the impact of prolonged fasting in adults with type 1 diabetes. The local ethics committee of the Medical University of Graz (Austria) approved the study protocol (30-238 ex 17/18), which was registered at the German Clinical Trials Register (DRKS00016148; DRKS.de). The study was conducted in conformity with the declaration of Helsinki and Good Clinical Practice. Before any trial related activities, potential participants were informed about the study protocol and participants gave their written informed consent.

### Eligibility Criteria

Eligibility criteria included a diagnosis of type 1 diabetes for longer than 12 months, age ≥ 18 years, treatment with exogenous insulin by means of MDI or continuous subcutaneous insulin infusion (CSII), c-peptide level ≤0.3 nmol/l, glycated hemoglobin (HbA_1c_) <80 mmol/mol (<9.5%) and a body mass index (BMI) 20─29.9 kg/m^2^. Individuals were not included if they experienced diabetic ketoacidosis or had a history of severe hypoglycemia requiring external assistance within the last 12 months, had a history of any life-threatening disease or any other condition that could influence the study procedures. Additionally, participants had to wear an intermittently scanned continuous glucose monitoring system (isCGM; FreeStyle Libre 1, Abbott, USA) for the assessment of interstitial glucose levels during the fasting period and hypoglycemia was verified by capillary blood glucose measurement.

### Assessment of Eligibility

Inclusion and exclusion criteria were assessed by a study physician at the screening visit, performed at least three days prior to the first fasting period. A venous blood sample was drawn for the assessment of HbA_1c_ and c-peptide levels and general health status was assessed by means of physical examination and determination of complete blood count and urine sample. Cardiovascular status was investigated *via* resting electrocardiogram and blood pressure measurements.

### Fasting Period

After inclusion in the study, participants were explained to record physical activity and episodes of hypoglycemia (<70 mg/dL; <3.9 mmol/L) and hyperglycemia (>270 mg/dL; 15.0 mmol/L) requiring treatment during the fasting period in a diary. Participants using MDI injected their regular basal insulin dose, as discussed in their regular Diabetes Outpatient Clinic visit. Those that were using CSII applied their regular basal insulin rate, however, if required, the basal rate was lowered by up to 25% if deemed necessary by the participants. The fasting period was defined as absolute avoidance of any caloric intake including caffeinated drinks and alcohol, although the consumption of tap water and soda was allowed ad libitum. The amount of consumed water was also recorded in a diary by the participants. The (first) short fasting period lasted 12 hours from 8 PM until 8 AM the next morning. The (second) prolonged fasting period had a duration of 36 hours starting at 8 PM. Both fasting periods were separated by at least one week and for both fasting periods, the participants were advised to apply the same amount of basal insulin either in the evening before, in the morning or both.

### Trial Visits

After each fasting period, participants attended the Clinical Research Facility for the assessment of anthropometric parameters, resting metabolic rate (Metamax 3B, Cortex, GER) and a bioelectrical impedance analysis (BIA; BIACORPUS RX 4004M, Medical Health Care GmbH, GER). For the resting metabolic rate, a face mask held in place by a nylon harness covered the participant’s nose and mouth. The mask was attached to a bidirectional digital turbine flow meter to measure the volume of inspired and expired air. A sample line between the turbine and analyzer unit determined O_2_ and CO_2_ content. A two point calibration procedure was conducted before each testing session according to the manufacturer’s guidelines (Calibration Manual 931-00-264/Revision a/2014-03-06, CORTEX Biophysik GmbH, Leipzig, Germany). Participants rested for a period of 20 minutes in the supine position before resting metabolic rate testing, during which breath by breath measurements were recorded for 30 minutes and stability of data were automatically selected by the system.

Afterwards, venous blood samples were drawn for the assessment of fasting glucose, insulin, cortisol, glucagon, beta-hydroxybutyrate and further metabolic markers. In addition, glucose, insulin, c-peptide, glucagon and beta-hydroxybutyrate levels were assessed over the course of the oral glucose tolerance test (OGTT): immediately before (T1), 15 min (T2), 30 min (T3), 60 min (T4), 120 min (T5), 180 min (T6) and 240 min (T7) after drinking the standardized 75 g carbohydrate drink (Glucoral, Germania Pharmazeutika, AUT). Immediately before both OGTTs, the same bolus insulin dose based on the regular carbohydrate-to-insulin ratio was injected. Capillary blood glucose measurements were performed for safety reasons in case of fast decreasing or increasing sensor glucose levels. OGTTs were discontinued early if the capillary blood glucose concentration dropped below 70 mg/dL (3.9 mmol/L) and immediately 15─30 gr carbohydrates were orally ingested by the participants.

### Outcome Measurements

We defined the change in 2 hours postprandial glucose during the OGTTs as primary outcome in this study. Additional outcomes included hypoglycemic events, glycemic patterns as assessed by isCGM and during the OGTT as well as body composition.

### Statistical Assessments

**Figure 1:**
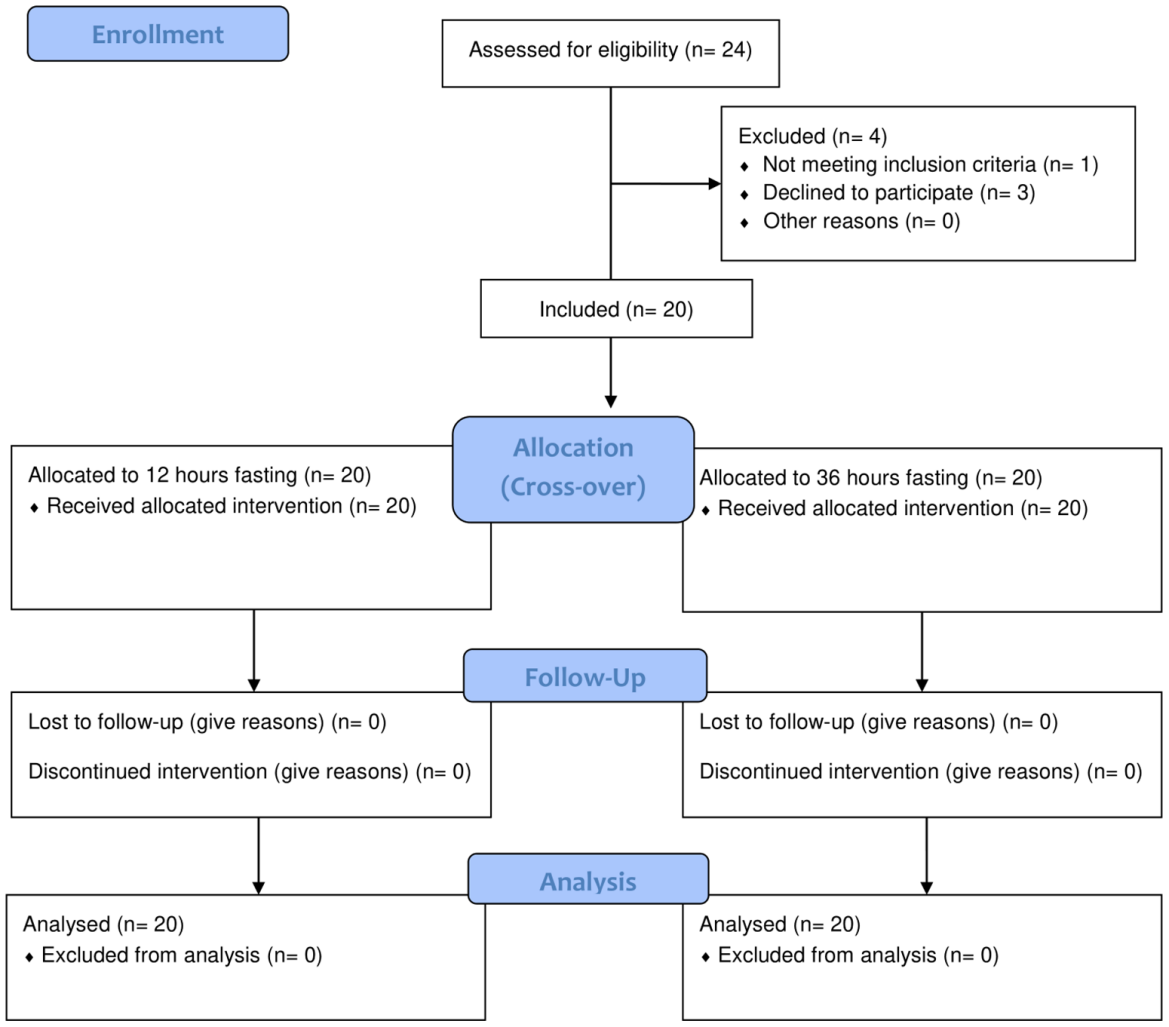
Study flow diagram.

All data were assessed for normal distribution by means of Shapiro-Wilk normality testing. Interstitial glucose levels during the fasting period were stratified for time below range level 2 (<54 mg/dL; <3.0 mmol/l), time below range level 1 (54─70 mg/dL; 3.0─3.9 mmol/L), time in range (70─180 mg/dL; 3.9─10.0 mmol/L), time above range level 1 (181─250 mg/dL; 10.1─13.9 mmol/L) and time above range level 2 (>250 mg/dL; >13.9 mmol/L). Singular post-fasting measurements and area under the curve (AUC; trapezoidal rule) in comparison of overnight vs. prolonged fasting were assessed by means of paired t-test or Mann-Whitney U test. Variables that were investigated over the course of the OGTT were compared between overnight vs. prolonged fasting *via* two-way ANOVA or mixed-model regressions (p ≤ 0.05). For the sample size estimation, we assumed a difference in the 2h-glucose during the OGTT of 20 ± 25 mg/dL (1.1 ± 1.4 mmol/L) between 12 hrs and 36 hrs fasting. Based on a paired t-test (two-sided, alpha 5%, power 90%), 19 participants were required to demonstrate the assumed difference. To compensate potential dropouts, 20 participants were included (**Figure 1**).

## Results

In total 20 individuals with type 1 diabetes (7 females) were included in the study with a mean ± SD age of 35 ± 11 years, BMI 24.8 ± 2.8 kg/m^2^, HbA_1c_ 54 ± 7 mmol/mol (7.1 ± 0.6%), diabetes duration 20 ± 11 years, total daily insulin dose 40 ± 14 IU. Eleven participants were using MDI therapy (6 insulin Aspart (Novo Nordisk, DEN), 4 insulin Lispro (Eli Lilly, USA), 1 faster insulin Aspart (Novo Nordisk, DEN); 5 insulin Degludec (Novo Nordisk, DEN), 6 insulin Detemir (Novo Nordisk, DEN) and 9 were using CSII therapy (2 insulin Lispro (Eli Lilly, USA), 7 insulin Aspart (Novo Nordisk, DEN).

### Glycemia During Fasting

Mean interstitial glucose levels were similar in comparison of the two fasting periods (12 hrs: 138 ± 35 mg/dL [7.7 ± 1.9 mmol/L] *vs.* 36 hrs: 130 ± 17 mg/dl [7.2 ± 0.9 mmol/L], p = 0.44). Coefficient of variation was numerically but not statistically lower for the short fasting period (12 hrs: 25 ± 10% *vs.*. 36 hrs: 31 ± 9%, p = 0.07). SD of interstitial glucose was not significantly different between both fasting periods (12 hrs: 27 ± 12 mg/dl [1.5 ± 0.7 mmol/L] *vs.* 36 hrs: 29 ± 10 mg/dL [1.7 ± 0.5 mmol/L], p = 0.41). When data were separated for day- (06:00 AM-12:00 PM) and night time (12:01 PM-05:59 AM), a lower mean interstitial glucose concentration was seen during the night time period for the prolonged fasting period (12 hrs: 139 ± 46 mg/dL [7.7 ± 2.6 mmol/L] *vs.* 36 hrs: 112 ± 22 mg/dL [6.2 ± 1.2 mmol/L], p = 0.05); furthermore, lower glycemic variability was observed during the prolonged fasting period (12 hrs: 27 ± 10 *vs.* 36 hrs: 16 ± 12%, p = 0.01). Other interstitial glucose parameters as given in **Table 1** remained similar between both trial arms (p>0.05).

**Table 1:** Comparison of glycemic ranges for 12 hrs vs. 36 hrs fasting (n=20).

Glycemic Range	12 hrs fasting	36 hrs fasting	p-value
**TAR 2 (>250 mg/dL; >13.9 mmol/L) (%)**	2 ± 5	1 ± 2	0.99
**TAR 1 (181─250 mg/dL; 10.1─13.9 mmol/L) (%)**	19 ± 22	13 ± 11	0.93
**TIR (70─180 mg/dL; 3.9─10.0 mmol/L) (%)**	72 ± 23	80 ± 14	0.77
**TBR 1 (54─70 mg/dL; 3.0─3.9 mmol/L) (%)**	5 ± 7	4 ± 3	0.98
**TBR 2 (<54 mg/dL; <3.0 mmol/l) (%)**	2 ± 5	2 ± 2	0.99

TAR 2, time above range 2; TAR 1, time above range 1; TIR, time in range; TBR 1, time below range 1; TBR 2, time below range 2 (n=20).

The rate of hypoglycemia per hour over the fasting period defined as levels below 70 mg/dL (3.9 mmol/L) was similar in comparison of both trial arms (12 hrs: 0.07 ± 0.06 *vs.* 36 hrs: 0.05 ± 0.03, p = 0.21) facing median and interquartile range interstitial glucose nadir of 60 mg/dL [48─68 mg/dL] (3.3 mmol/L [2.7─3.8 mmol/L] for the overnight fasting period and 63 mg/dl [58─68 mg/dL] (3.5 mmol/L [3.2─3.8 mmol/L] for the prolonged fasting period (p = 0.35). 59% of episodes of hypoglycemia required supplemental carbohydrates during the 12 hrs fasting period (18 gram [15─24]) *vs.* 71% during the 36 hrs fasting period (25 gram [15─27] (p = 0.55). Remaining episodes of hypoglycemia, mainly occurring during the night time period and were endogenously regulated. Potentially prandial bolus insulin associated hypoglycemia (+3 hrs of last dosing) accounted for 17% of all hypoglycemic episodes in both groups. Those with a higher dose of basal insulin/basal insulin rate had a significantly higher absolute number of hypoglycemic episodes when compared to those with a lower dose during the 36 hrs fasting period (total daily basal insulin dose (TDBD) <0.25 IU/kg: 1.33 ± 0.86 *vs.* TDBD >0.25 IU/kg: 2.5 ± 0.85, p = 0.008), but not during the 12 hrs fasting period (TDBD <25 IU/kg: 1.0 ± 1.8 *vs.* TDBD >25 IU/kg: 1.3 ± 0.95, p = 0.53). For both trial arms, the basal insulin rate per hour was similar for CSII (12 hrs fasting: 0.92 ± 0.18 IU/hr *vs.* 36 hrs fasting: 0.88 ± 0.24 IU/hr, p = 0.33) and identical for MDI (for both trial arms: 0.82 ± 0.22). No bolus insulin dose was applied in any of the participants during the fasting periods. For both trial arms, all participants were following the fasting regimen, except when carbohydrates were given to treat hypoglycemia. Time spent in the pre-specified glycemic ranges is given in **Table 1**.

### Effects of Prolonged Fasting on Resting Metabolic Rate and Anthropometry

The resting metabolic rate was similar in comparison of both trials arms (12 hrs fasting: 2191 ± 357 kcal/day *vs.* 2186 ± 349, p = 0.92), while the respiratory exchange ratio was lower for prolonged fasting (12 hrs fasting: 0.87 ± 0.05 (/) *vs.* 36 hrs: 0.82 ± 0.03 (/), p = 0.001) accompanied by higher fat oxidation (12 hrs fasting: 90 ± 40 g/day *vs.* 36 hrs fasting: 130 ± 35 g/day, p < 0.001) and lower carbohydrates oxidation following prolonged fasting (12 hrs fasting: 305 ± 98 g/day *vs.* 36 hrs fasting: 215 ± 63, p = 0.007). Protein metabolism remained unaffected by prolonged fasting (12 hrs fasting: 25 ± 4 g/day *vs.* 36 hrs fasting: 25 ± 4, p = 0.63). Body weight (76.7 ± 13.5 kg *vs.* 75.4 ± 13.4 kg, p = 0.0002) and BMI (24.6 ± 2.8vs. 24.2 ± 2.8 kg/m2; p = 0.0001) following the prolonged fasting period were significant lower when compared against overnight fasting. Fat mass (p = 0.26), free fat mass (p=0.79), total body water (p = 0.46) and body cell mass (p = 0.73) were similar after both fasting periods.

### Effects of Prolonged Fasting on Metabolic Markers

Significant differences were found in comparison of post-12 hrs fasting *versus* post-36 hours fasting for uric acid (p < 0.001), bilirubin (p = 0.002), triglycerides (p < 0.001), VLDL (p = 0.011), serum iron (p = 0.040), ferritin (p = 0.01), transferrin saturation (p = 0.02) and leptin (p = 0.006) which can be found in **Table 2**.

**Table 2:** Effects of prolonged fasting on metabolic markers (n = 20).

Glycemic Range	12 hrs fasting	36 hrs fasting	p-value
**Uric acid (mg/dL)**	4.12 ± 1.02	4.97 ± 1.10	<0.001
**Bilirubin (mg/dL)**	0.90 ± 0.76	1.33 ± 1.17	0.002
**Triglycerides (mg/dL)**	64 ± 18	80 ± 28	<0.001
**Cholesterol (mg/dL)**	195 ± 32	201 ± 34	0.220
**HDL (mg/dL)**	73 ± 18	71 ± 20	0.357
**LDL (mg/dL)**	102 ± 34	109 ± 32	0.140
**VLDL (mg/dL)**	14 ± 4	17 ± 4	0.011
**Serum-Iron (µg/dL)**	125 ± 54	101 ± 35	0.040
**Ferritin (ng/mL)**	115 ± 67	135 ± 82	0.010
**Transferrin (g/L)**	2.3 ± 0.6	2.3 ± 0.5	0.140
**Transferrin saturation (%)**	42 ± 22	32 ± 13	0.020
**Gastrin (pg/mL)**	23 ± 11	23 ± 8	0.97
**Leptin (ng/mL)**	3.47 ± 3.25	2.42 ± 2.46	0.006

### Effects of Prolonged Fasting on Metabolic and Hormonal Markers During OGTT

**Figure 2:**
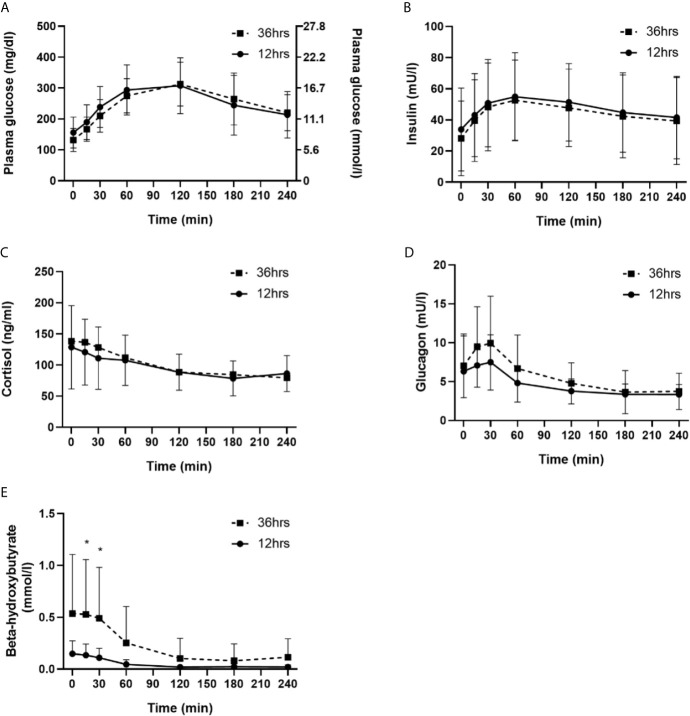
Comparison of 12 hrs fasting *vs.* 36 hrs fasting for plasma glucose **(A)**, insulin **(B)**, cortisol **(C)**, glucagon **(D)** and beta-hydroxybutyrate **(E)**. * indicates significant difference for the specific time point n comparison of both trial arms (n = 20).

OGTTs were discontinued due to hypoglycemia three times in three people in each trial arm. The median [interquartile range] of the 120 min OGTT plasma glucose AUC were similar in comparison of both fasting regimes (12 hrs fasting 32798 [24791─38831] vs. 36 hrs fasting [30146 [26361─33154]; p = 0.21). Mean 120 min plasma glucose levels were also similar in comparison of both trial arms (12 hrs fasting 308 ± 91 mg/dL [17.1± 5.1 mmol/L] vs. 36 hrs fasting 313 ± 71 mg/dL [17.4 ± 3.9 mmol/L; p = 0.73). For the OGTTs, exactly the same dose of bolus insulin was applied (6 ± 2 IU) in both trial arms. Over the course of the OGTT, plasma glucose levels significantly increased for both trial arms (p < 0.001), following a similar course (p = 0.04). The course of plasma glucose, insulin, glucagon, beta-hydroxybutyrate and cortisol in comparison of both trial arms is detailed in **Figure 2**. Three episodes of hypoglycemia occurred after the overnight fasting during the OGTT (mean glucose nadir 64 ± 5 mg/dL [3.6 ± 0.3 mmol/L]) and three episodes of hypoglycemia occurred after the prolonged fasting during the OGTT (mean glucose nadir 65 ± 6 mg/dL [3.6 ± 0.3 mmol/L]) - those three people had also an episode during the overnight fasting period (comparison of hypoglycemic events between groups, p = 0.50).

### Differences in MDI Versus CSII Therapy

During the 12 hrs fasting period, participants using MDI therapy spent more TIR when compared to those using CSII therapy (MDI 83 ± 15% vs. CSII 62 ± 25%; p = 0.006), which was not seen during the 36 hrs fasting period (MDI 78 ± 17% *vs.* CSII 82 ± 11%; p = 0.74). Remaining glycemic ranges, risk of hypoglycemia and insulin therapy were not significantly different in comparison of the type of therapy (p > 0.05). During the OGTT for both after the 12 hrs (p = 0.0002) and 36 hrs fasting period (p < 0.0001), the MDI group had higher insulin levels when compared to the CSII group. Remaining markers assessed during the OGGT where not significantly different in comparison of groups (p > 0.05).

## Discussion

This is the first study investigating the glycemic effects and safety of a 36 hrs prolonged fasting period in adults with type 1 diabetes. Out of our results it might be concluded that prolonged fasting up to 36 hrs is feasible accompanied by a low risk of dysglycemia in people under good glycemic control. A similar conclusion has be drawn in a recent study when a multimodal fasting intervention was performed over a period of 7 days in adults with type 1 diabetes (13). Furthermore, Berger et al. showed that such a prolonged fasting period can maintain euglycemia while β-hydroxybutyrate levels were elevated by up to 2.8 mmol/L and bodyweight as well as BMI decreased and were maintained for up to 4 months after the intervention (13).

As concluded in a previous review, insulin resistance to exogenous insulin is deteriorating over the course of type 1 diabetes, which was found to be associated to some extent to bodyweight (17). In the transition from the Diabetes Control and Complications Trial to the Epidemiology of Diabetes Interventions and Complications study, it was shown that excess weight gain was associated with insulin resistance (18). Taking into account that in people with type 1 diabetes exogenous insulin is continuously circulating that is inversely related to rates of lipolysis, safe and feasible strategies need to be defined for bodyweight and insulin resistance management. Even a healthy lifestyle consisting of physical activity and exercise (19, 20) as well as a balanced diet (21), might not solely be sufficient for losing body weight in type 1 diabetes and therefore people are using alternative strategies with significant caloric restriction, requiring withholding bolus insulin doses. Therefore, a solid research basis is required for future patient counselling.

Intriguingly, in our study during the prolonged fasting, the basal insulin rate for CSII was only lowered by ~4% that was sufficient to prevent hypoglycemia. This finding differs to recent recommendations, in which it was detailed that people with type 1 diabetes should reduce the basal insulin rate initially by 10% to an extent of as much as 90% towards the end of the fasting day (22). However, given the nature of our well-controlled and safe study design and the single fasting episode only, a direct comparison to these recommendations might be difficult. Therefore, it is important to point out, that if basal insulin rate is reduced to a greater extent, not only blood glucose should be monitored but also ketone levels (23). In line with previous research (24), fasting induced a significant increase in serum uric acid levels that might be evoked by a decrease in uric acid excretion by the kidney in the presence of elevated ketones which were significantly higher (beta-hydroxybutyrate of 0.54 mmol/l) after the prolonged fasting period than after 12 hours (0.15 mmol/). Total serum bilirubin concentration did rise significantly, as it was observed previously (25), however, the mean value was just above the normal range. While gastrin, a hormone that increases the release of gastric acid, remained unchanged by prolonged fasting, leptin, a hormone that regulates the sensation of hunger, significantly decreased, which is in line with a previous study performed in healthy individuals (26).

For both, during the fasting period as well as with the first dosing after the fasting, prolonged fasting can be safely performed in people with well-controlled type 1 diabetes. Numerically, less episodes of hypoglycemia were observed during the prolonged fasting period when compared against the overnight fasting. It can be hypothesized that our participants did more thoroughly monitor their glucose levels before and during the fasting, yielding such a low number of hypoglycemia by consuming carbohydrates. In line with this finding, TIR was not different between overnight fasting and prolonged fasting. From a physiological point of view, one might assume that with the first dosing after a prolonged fasting period the carbohydrates-to-bolus insulin ratio might be lowered due to hepatic glycogen depletion during fasting or potentially *via* an increased insulin sensitivity. Intriguingly, we did observe the same glycemic excursions during the OGTT in both groups and three episodes of hypoglycemia occurred during the OGTT following prolonged and 12 hours fasting. This finding suggests that people with type 1 diabetes do not need to adapt their bolus insulin dose with the first meal after a prolonged fasting period. In line with the low risk of hypoglycemia in comparison of both trial arms, hormones and metabolic markers showed similar patterns during the OGTT, except beta-hydroxybutyrate. However, these physiologically elevated ketone levels reflect the shift to lipolysis during fasting (27).

Our study is not without limitations: since this was the first study assessing the safety and glycemic effects of prolonged fasting, our study participants were not randomized to the sequence of the 12 hrs and 36 hrs, therefore 12 hrs fasting periods were always followed by the 36 hours period. In addition, in case of a hypoglycemic event, participants administered carbohydrates and strictly speaking the fasting period ended at this time point. However, this administration was critical for safety reasons. Furthermore, only participants running on CSII were allowed to lower the basal insulin rate but not those with MDI. Notwithstanding, within the group of participants on CSII the basal insulin rate was similar for both trial arms. While this is important data on prolonged fasting in people with type 1 diabetes, we appreciate that from a single 36 fasting period one cannot extrapolate to the safety of intermittent fasting over a longer period of time in this population, an aspect that clearly needs further attention and investigation. Furthermore, the sample size for assessing the safety and efficacy was in general small but can serve as the basis for sample size estimations in future, larger fasting studies. Additionally, we did not assess the status of the menstrual cycle that might also have an impact on the results. In addition, it clearly needs to be pointed out that an OGTT does not reflect normal nutrition habits after a fasting period; so our procedure was artificially mimicking the first carbohydrate intake after prolonged fasting. For future studies, it is also urgently needed to assess blood ketone levels not only at the end of the fasting period but also continuously during the fasting period.

## Conclusions

Our proof-of-concept study showed that people with type 1 diabetes under good glycemic control might be able to safely perform a prolonged fasting period with a limited risk of severe dysglycemia. However, we performed a study with a single fasting episode only, which cannot be extrapolated to intermittent fasting interventions of longer duration. Hence, future studies over a more extended period of time are required to detail the entire spectrum of potentially beneficial effects of interventional fasting in people with type 1 diabetes.

## Data Availability Statement

The raw data supporting the conclusions of this article will be made available by the authors, without undue reservation upon request.

## Ethics Statement

The studies involving human participants were reviewed and approved by the local ethics committee of the Medical University of Graz (Austria) (30-238 ex 17/18). The patients/participants provided their written informed consent to participate in this study.

## Author Contributions

OM, ME, NT, PP, and HS designed and performed the study, interpreted data, and contributed to discussions. OM, HY, NG, and FAbe drafted the manuscript and/or performed statistical analysis. OM, ME, AM, NT, HY, FAbe, FAbb, PP, FA, AO, TP, HK, CS, MB, TN, MH, and HS performed the study. All authors contributed to the article and approved the submitted version.

## Funding

This study was supported by the Austrian Science Fund (KLIF-851).

## Conflict of Interest

The authors declare that the research was conducted in the absence of any commercial or financial relationships that could be construed as a potential conflict of interest.
